# Length-weight relationship and condition factor of Nile tilapia ( *Oreochromis niloticus*) fed diets supplemented with guava and star gooseberry leaf extract

**DOI:** 10.12688/f1000research.145369.2

**Published:** 2024-10-23

**Authors:** Manoj Tukaram Kamble, Krishna Rugmini Salin, Balasaheb Ramdas Chavan, Seema Vijay Medhe, Kim D. Thompson, Nopadon Pirarat

**Affiliations:** 1 Aquaculture and Aquatic Resources Management (AARM), Department of Food, Agriculture and Bioresources, School of Environment, Resources, and Development, Asian Institute of Technology, Khlong Nueng, Pathum Thani, 12120, Thailand; 2 Centre of Excellence in Wildlife, Exotic and Aquatic Animal Pathology, Faculty of Veterinary Science, Chulalongkorn University, Bangkok, 10330, Thailand; 3 Department of Aquaculture, College of Fisheries, Ratnagiri, Maharashtra, 415629, India; 4 Moredun Research Institute, Pentlands Science Park, Penicuik, Scotland, EH26 0PZ, UK

**Keywords:** Nile tilapia, length-weight relationship, condition factor, specific growth rate, guava, star gooseberry

## Abstract

**Background:**

Nile tilapia ( *Oreochromis niloticus*) is predominant cultured species in aquaculture. However, there is a scarcity of literature regarding relationship between guava and star gooseberry leaf extract and the condition factor. Thus, the present study aims to investigate the effect of guava and star gooseberry leaf extract-supplemented diets on the specific growth rate, length-weight relationship, and condition factor of Nile tilapia.

**Methods:**

Six hundred and thirty Nile tilapia (8.7±0.4 g) were randomly distributed among twenty-one tanks (30 fish per tank) within a recirculation system. Over a 60-day period, the fish were fed diets supplemented with 5g/Kg and 10g/Kg of guava leaf extract (GLE-5, GLE-10), star gooseberry leaf extract (SGLE-5, SGLE-10), and a mixture of both (MxLE-5, MXLE-10). Subsequently, specific growth rate, length-weight relationship, and condition factor were determined.

**Results:**

After 60 days, the specific growth rate was significantly higher in all the GLE, SGLE, and MxLE groups compared to the control group. The final lengths and weights differed significantly both in the control group and all the GLE, SGLE, and MxLE groups. The analysis of the regression equation indicated a positive correlation (r = 0.970, 0.977, 0.976, 0.974, 0.974, 0.974, and 0.969) between the length and weight of Nile tilapia in the control group and in all the GLE, SGLE, and MxLE groups. The regression exponent “ *b*” values in all the GLE, SGLE, and MxLE groups were >3, indicating a positive allometric growth pattern in Nile tilapia compared to the control ( *b*=2.866), which exhibited a negative allometry. The final condition factor values did not differ significantly in either the control group or any of the plant extract groups.

**Conclusions:**

Nile tilapia exhibited positive allometric growth patterns and maintained good health when fed with GLE, SGLE, and MxLE groups. Therefore, these plant extracts demonstrate suitability for commercial Nile tilapia production.

## Introduction

Nile tilapia ( *Oreochromis niloticus*) is recognized as an important species in aquaculture within tropical and subtropical regions, owing to its faster growth, heightened productivity, and notable resistance to diseases. ^1^^–^^4^ In 2020, the global production of Nile tilapia nearly reached 4.9 million metric tons (Mmt), and the continuous growth is attributed to the robust worldwide demand. ^5^ Furthermore, technological advancements with high stocking densities play a significant role in the occurrence of disease outbreaks, resulting in substantial economic losses and impeding the sustainable progression of tilapia culture globally. ^6^

The incorporation of plant extracts into aquafeed is significant in finfish aquaculture ^7^ because the indiscriminate use of antibiotics has led to the development of drug-resistant bacteria. ^8^^,^^9^ Plant extracts can serve as cost-effective and environmentally sustainable prophylactic or antimicrobial agents. Guava ( *Psidium guajava*) leaf (GLE) are rich in various phenolic and flavonoid compounds ^10^ and possess antimicrobial, ^11^ antiviral, ^12^ and antioxidant ^13^ activities. GLE have been employed as supplements in fish feed to boost the growth, immune response, and protection against diseases for various fish species. ^14^^–^^20^ Star gooseberry ( *Phyllanthus acidus*) leaf (SGLE) contain flavonoids, kaempferol, hypogallic acid, caffeic acid, and phenolic compounds. ^21^^,^^22^ SGLE exhibited antioxidant, anti-inflammatory and antimicrobial activities. ^23^^,^^24^ It is also employed as a dietary supplement to augment the immune response of fish ^9^^,^^25^ and chickens. ^26^

The length-weight relationship (LWR) plays an essential role in estimating the biomass of diverse fish populations. ^27^ Additionally, LWR is valuable for assessing fish condition and growth patterns. ^28^ The condition factor (CF) contributes to comprehending the development and adaptability of fish. ^29^ A heightened condition factor indicates the robust health and positive growth of the fish. ^30^ Hence, the condition factor can be employed to assess the feeding activity of a species, indicating its effective utilization of available feeding sources. ^27^ Previous studies have investigated the length-weight relationship and feeding habits of tilapia. ^27^^,^^30^^–^^33^ Unfortunately, there is a scarcity of literature regarding relationship between guava and star gooseberry leaf extract and the condition factor. Thus, the present study aims to investigate the effect of guava and star gooseberry leaf extract-supplemented diets on the specific growth rate, length-weight relationship, and condition factor of Nile tilapia.

## Methods

### Chemicals

The analytical grade chemicals utilized for the determination of Ammonia, Nitrite, and Nitrate from the experimental tanks included Sodium Hypochlorite 10% (Commercial, U&V Holding (Thailand) CO., LTD), Hydrochloric acid, 37% (Fisher Scientific UK), Sodium hydroxide (Pine Chemical, Finland), Phenol, 99+% (Fisher Scientific UK), Sulfanilamide (Acros Organics Bvba, Belgium), N-(1-Naphthyl) ethylenediamine dihydrochloride (Alfa Aesar, USA), Cadmium granular (ALDRICH Chemical, USA), Manganese (II) Sulphate H2O, Ammonium Chloride, Sodium Nitrite, Copper (II) sulfate, and Ethylenediamine Tetra Acetic acid (Daejung Chemicals and Metals, South Korea), as well as Sodium Tetraborate and Potassium Nitrate (KEMAUS, Australia) (Underlying Data). ^34^

### Preparation of guava and star gooseberry leaf aqueous extracts

The guava ( *P. guajava*) and star gooseberry ( *P. acidus*) leaf powder were received from All-Season Herbs Pvt. Ltd., Bangalore, India and the aqueous extract was prepared. ^35^ Briefly, distilled water (100 mL) was mixed with leaf powder (10 g) and homogenized using an orbital shaker (Hsiangtai D500) at 100 rpm for 20 h at room temperature (RT). The resultant mixture underwent centrifugation at 8,000 rpm for 15 min at RT. A rotavapor (BÜCHI R-200/205) at 35 °C used to evaporate the supernatant and continue drying for 48 h in a hot air oven at 50 °C. The dried samples obtained were placed in bottles and kept refrigerated at 4°C until further use.

### Ethical statement

This study was conducted under the project entitled ‘Application of Phytobiotic Supplementation to Improve Disease Resistance of Nile Tilapia ( *Oreochromis niloticus*)’ and funded by the Social Justice and Special Assistance Department, Government of Maharashtra, India, and Second Century Fund (C2F) Postdoctoral Fellowship, Chulalongkorn University, Bangkok, Thailand. This study was carried out in strict accordance with the recommendations of the Asian Institute of Technology (AIT), Thailand, and the protocol was approved by the Local Ethical Committee for Experiments on Animals of the AIT, Thailand (Project Number: AIT-AQ-18-01, May 2018–April 2019; April 29, 2018). Additionally, according to Thailand Legislation, the Nile tilapia were not categorized as a protected species. The research was carried out at the Laboratory of the Aquaculture and Aquatic Resources Management (AARM, Asian Institute of Technology (AIT), Thailand, over 60 days from February to March, 2019. This study is reported in line with the ARRIVE guidelines. A total of 630 fish were used in the experiment, with 90 fish allocated to each treatment. Each treatment comprised 30 fish per replicate, totaling three replicates. Prior to sampling, the fish underwent a 12-hour fasting period to empty their digestive tracts. In this study, to reduce stress on the experimental animals, the animals housed in the containers were gently anesthetized using 1 mL of clove oil per 10 L of water for a period of 2–3 min, after which their weights were measured. Following this, the fish were returned to their designated glass tanks according to their treatment and replication groups.

### Fish

Nile tilapia monosex fingerlings (8.7 ± 0.4 g and 8.0 ± 1.0 cm) were obtained from a GMP Certified fish farm, Ayutthaya Province, Thailand. The research was conducted at the laboratory of Aquaculture and Aquatic Resources Management (AARM), Asian Institute of Technology (AIT), Thailand. For a period of 15 days, the fish were acclimatized in three fiberglass tanks (500 L) before the commencement of the experiment. Fish were fed pelleted feed twice daily at 4% of their body weight. Following the acclimatization period, the fish were randomly distributed among twenty-one 150-L glass tanks (30 fish per tank), all integrated into a recirculation system.

### Preparation of experimental diets supplemented with guava and star gooseberry leaf aqueous extracts

Plant extracts (10 mg), dissolved in 1 mL distilled water, were mixed with commercial feed ((Charoen Pokphand (CP)-7710), and spaghetti-like strands were prepared using a mincer (MITSUYAMA YC80B-4). Pellets approximately 5 mm in length were formed and dried for 24 h at 50 °C in a hot air oven. The dried pellets were then stored at 4 °C until the end of the experiment. The proximate composition of the commercial diet, including moisture, ash, crude lipid, and crude protein, was 7.6%, 8.2%, 6.5%, and 30.5%, respectively.

### Experimental design

In this study, a completely randomized design was employed, comprising seven experimental treatments: *5g/kg and 10g/kg of guava leaf extract (GLE-5, GLE-10), star gooseberry leaf extract (SGLE-5, SGLE-10), and a mixture of both (MxLE-5, MXLE-10).* The mixed diets (MxLE) were prepared with an equal proportion (1:1) of GLE and SGLE extracts. The control group, on the other hand, was fed a diet without plant extract supplementation. Fish were fed twice daily at 4% of their body weight. Prior to sampling, the fish underwent a 12-hour fasting period to empty their digestive tracts. In this study, to reduce stress on the experimental animals, the animals housed in the containers were gently anesthetized using 1 mL of clove oil per 10 L of water for a period of 2–3 min, after which their weights were measured. Following this, the fish were returned to their designated glass tanks according to their treatment and replication groups. The specific growth rate, length-weight relationship, and condition factor were determined after 60 days of feeding.

### Water quality parameters

During the experiment, a Eutech Cyberscan PC300 multi-parameter apparatus was employed to measure daily water quality parameters, including temperature, pH, and dissolved oxygen (DO). The weekly concentrations of ammonia-nitrogen (NH _3_-N) were assessed using Phenate or indophenol method, ^36^ nitrite-nitrogen (NO _2_-N) concentrations were quantified through direct spectrophotometric assay, ^36^ and nitrate-nitrogen (NO _3_-N) levels were determined employing cadmium reduction method. ^36^

### Specific growth rate of Nile tilapia fed diets supplemented with GLE, SGLE and MxLE

The specific growth rate ^37^ was evaluated after 60 days of feeding trials using the following equation

specific growth rate(SGR,%/day)=(100×(lnFinal weight(g)–lnInitial weight(g))/(experimental days(T))

### Length-weight relationship (LWR) of Nile tilapia fed diets supplemented with GLE, SGLE and MxLE

Length-weight relationship (LWR) ^38^ between the total length and body weight was estimated using the following formula

$$W={\mathrm{aL}}^{b}$$

Where W is body weight of fish (g), L is the total length (cm), a is the exponent denotes the rate of weight change with length (constant), and b is the slope representing the weight of one unit length. These values are estimated through the linear regression equation, which is transformed by applying the natural logarithm (Log) to both sides.

LogW=Loga+b.LogL

Where b = 3, growth pattern is isometric. However, when b is greater or less than 3, an allometric pattern emerges. This allometric pattern can be positive, indicating increase in length relative to body thickness, or negative, signifying an increase in length relative to body thinness. ^28^

### Relative condition factor (K _n_) of Nile tilapia fed diets supplemented with GLE, SGLE and MxLE

The health condition of the tilapia was assessed by evaluating relative condition factor (K _n_) in all the treatments.

$$K_{n}=W_{o}/W_{c}$$

where W _o_ = observed weight, and W _c_ = calculated weight. ^39^ When K _n_ is greater than 1, it indicates favorable growth conditions for the fish. Conversely, when K _n_ is less than 1, it suggests that the organism is in poorer growth condition compared to an average individual of the same length.

### Data analysis

The results were presented as means ± SE, and statistical analysis was conducted using IBM SPSS Statistics software (SPSS, Inc., Version 29). The homogeneity of data was determined using Levine’s test. The impact of plant extract treatments on specific growth rate was assessed through one-way analysis of variance (ANOVA), followed by Tukey post-hoc tests for multiple comparisons. An independent t-test was used to determine the significant difference between the initial and final length and weight of Nile tilapia with plant extract treatments. Linear regression analysis was employed to evaluate the length-weight relationship. The Pearson correlation matrix was used to analyze relationship between water quality and fish biometric parameters. A significance level of *p* < 0.05 was considered for statistical significance.

## Results

### Water quality parameters

Water quality parameters such as Temperature, pH, DO, NH _3_N, NO _2_N, and NO _3_N, did not show significant difference among the plant extract treatments and control during the experiment ( Table 1). In all treatments, the average values for temperature, pH, DO, NH _3_N, NO _2_N, and NO _3_N were 29.6 °C, 7.47, 5.49 mg/L, 0.15 mg/L, 0.13 mg/L, and 0.23 mg/L, respectively. Raw data are available as underlying data (Tables 1-6). ^34^

**Table 1.** Water quality parameters of Nile tilapia fed diets supplemented with plant extracts.

	Control	GLE-5	GLE-10	SGLE-5	SGLE-10	MxLE-5	MxLE-10
Temp (°C)	30.6±0.15	30.7±0.16	30.8±0.16	30.7±0.17	30.8±0.16	30.8±0.16	30.5±0.16
pH	7.39±0.02	7.16±0.02	7.18±0.01	7.31±0.03	7.21±0.02	7.26±0.02	7.27±0.02
DO (mg/L)	4.69±0.05	4.15±0.05	4.01±0.09	4.57±0.15	4.45±0.06	4.06±0.06	4.54±0.05
NH _3_N (mg/L)	0.13±0.01	0.18±0.01	0.18±0.01	0.14±0.01	0.17±0.01	0.21±0.01	0.16±0.01
NO _2_N (mg/L)	0.07±0.01	0.09±0.01	0.11±0.01	0.13±0.01	0.12±0.01	0.08±0.01	0.09±0.01
NO _3_N (mg/L)	0.27±0.01	0.24±0.01	0.23±0.01	0.25±0.01	0.26±0.01	0.24±0.01	0.25±0.01

Temp: Temperature; DO: dissolved oxygen; NH _3_N: Ammonia-Nitrogen; NO _2_N: Nitrite-Nitrogen; NO _3_N: Nitrate-Nitrogen; GLE-5: 5g/kg of guava leaf extract; GLE-10: 10g/kg of guava leaf extract; SGLE-5: 5g/kg of star gooseberry leaf extract; SGLE-10: 10g/kg of star gooseberry leaf extract; MxLE-5: 5g/kg of mixture of GLE and SGLE; MxLE-10: 10g/kg of mixture of GLE and SGLE.

### Specific growth rate

**Figure 1.**
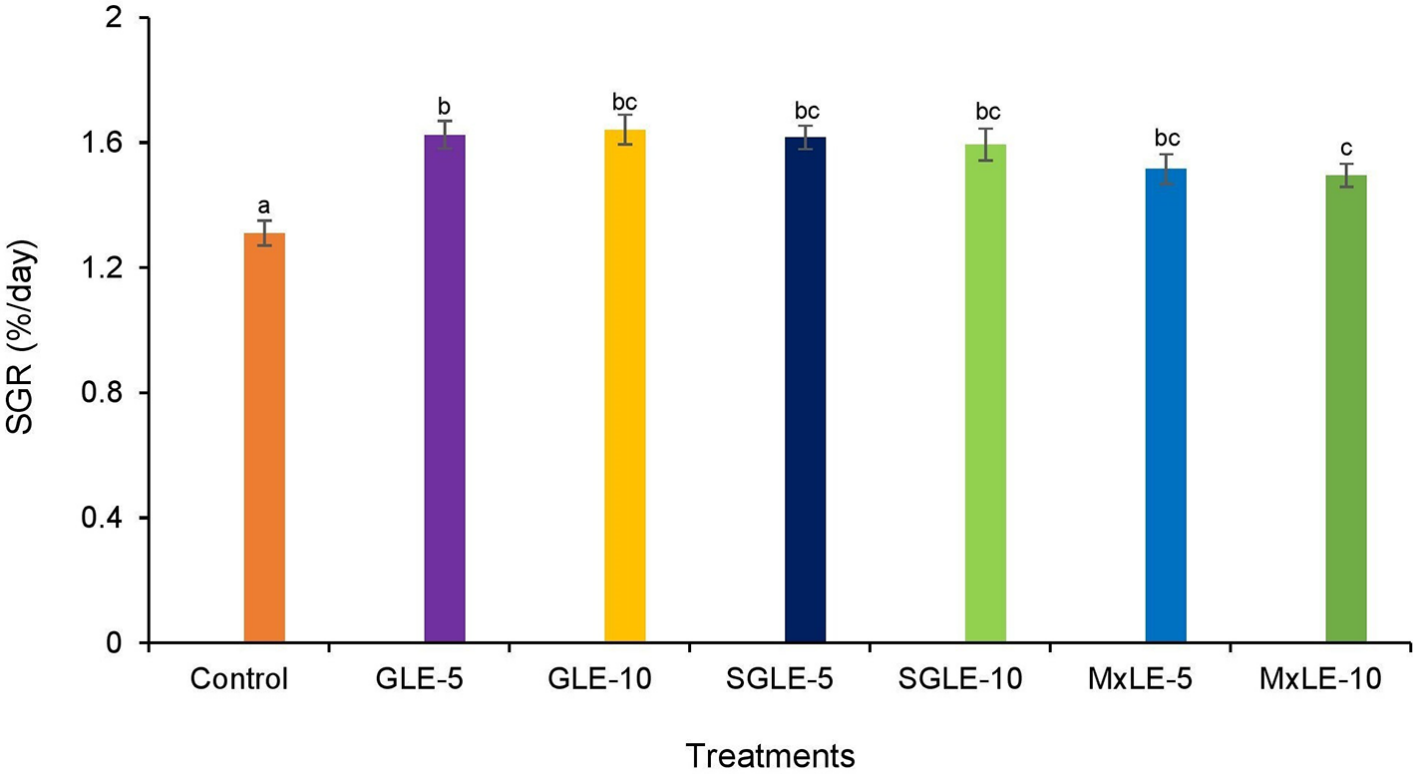
Specific growth rate of Nile tilapia fed diets supplemented with plant extracts. Results with the means of three replicates ± SE; Different superscripts in the bar indicate significant difference ( *p* < 0.05).

The survival rate was higher than 95% in all treatments. The specific growth rate was significantly higher in all plant extract treatments compared to the control group. GLE-10 exhibited a higher SGR, while MxLE-10 showed a lower SGR in the plant extract treatments ( Figure 1). Raw data are available as underlying data (Table 7). ^34^

### Length-weight relationship and growth pattern

The lengths were significantly different at the end of the experimental period in the control ( *t*(148)=-30.327, *p*=0.01), GLE-5 ( *t*(148)=-37.193, *p*=0.01), GLE-10 ( *t*(148)=-32.297, *p*=0.01), SGLE-5 ( *t*(148)=-37.552, *p*=0.01), SGLE-10 ( *t*(148)=-28.491, *p*=0.01), MxLE-5 ( *t*(148)=-31.824, *p*=0.01), and MxLE-10 ( *t*(148)=-38.139, *p*=0.01) groups ( Table 2). Additionally, the weights were significantly different at the end of the experimental period in the control ( *t*(148)=-31.206, *p*=0.01), GLE-5 ( *t*(148)=-38.611, *p*=0.01), GLE-10 ( *t*(148)=-32.694, *p*=0.01), SGLE-5 ( *t*(148)=-37.627, *p*=0.01), SGLE-10 ( *t*(148)=-30.032, *p*=0.01), MxLE-5 ( *t*(148)=-32.800, *p*=0.01), and MxLE-10 ( *t*(148)=-38.197, *p*=0.01) groups.

**Table 2.** Length-weight relationship, growth pattern, and condition factor of Nile tilapia fed diets supplemented with plant extracts.

	Control	GLE-5	GLE-10	SGLE-5	SGLE-10	MxLE-5	MxLE-10
N	75	75	75	75	75	75	75
L _Min-Max (_cm)	11.6-18.5	11.2-19.8	12.0-20.1	12.0-19.6	11.9-20.5	12.5-19.5	12.4-19.1
W _min-max_ (g)	25.0-106.0	24.0-145.0	28.0-157.0	29.6-132.0	28.8-156.0	30.0-134.0	31.0-128.0
*a*	-1.612	-1.797	-1.880	-1.853	-1.758	-1.917	-1.815
*b*	2.866	3.023	3.101	3.067	3.003	3.135	3.036
SE ( *b*)	0.085	0.077	0.081	0.083	0.082	0.085	0.091
CI ( *b*)	2.698-3.035	2.869-3.177	2.941-3.262	2.901-3.232	2.839-3.167	2.965-3.304	2.855-3.218
r	0.970	0.977	0.976	0.974	0.974	0.974	0.969
R ^2^	0.940	0.955	0.953	0.949	0.948	0.949	0.939
*p*	0.001	0.001	0.001	0.001	0.001	0.001	0.001
t-test sig	0.001	0.001	0.001	0.001	0.001	0.001	0.001
Growth behavior	Negative allometry	Positive allometry	Positive allometry	Positive allometry	Positive allometry	Positive allometry	Positive allometry
K _n_	1.004	1.002	1.003	1.003	1.004	1.003	1.003
Min-Max	0.865-1.177	0.886-1.178	0.824-1.097	0.840-1.282	0.817-1.162	0.857-1.136	0.822-1.162
SE	0.010	0.008	0.008	0.010	0.011	0.009	0.009

N: sample size; L: length (cm); W: weight (g); Min: minimum; Max: maximum; *a*: intercept; *b*: slope of the equation; SE: standard error; CI ( *b*): confidence intervals of *b*; r ^2^: coefficient of determination; *p*: significance of the regression, with *p* significant at <0.05; K _n_: relative condition factors. Significance was determined by the *t*-test to verify if *b* was significantly different from the consensus ( *b* = 3). The growth behavior was based on *b.* GLE-5: 5g/kg of guava leaf extract; GLE-10: 10g/kg of guava leaf extract; SGLE-5: 5g/kg of star gooseberry leaf extract; SGLE-10: 10g/kg of star gooseberry leaf extract; MxLE-5: 5g/kg of mixture of GLE and SGLE; MxLE-10: 10g/kg of mixture of GLE and SGLE.

The analysis of the regression equation indicated a positive correlation (r = 0.970, 0.977, 0.976, 0.974, 0.974, 0.974, and 0.969) between the length and weight of Nile tilapia fed control, GLE-5, GLE-10, SGLE-5, SGLE-10, MxLE-5, MxLE-10 diets, respectively.

**Figure 2.**
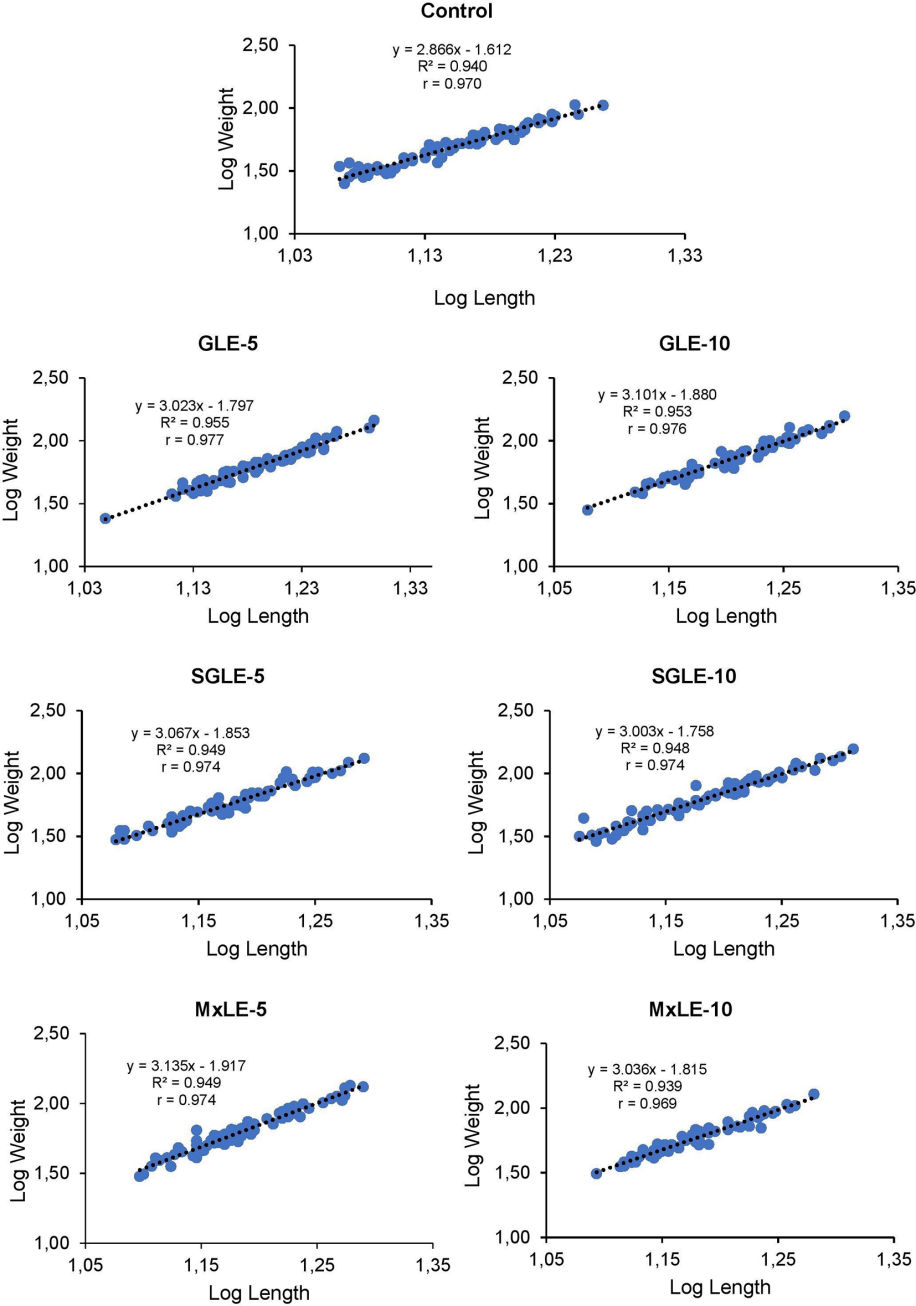
Final logarithmic length-weight relationship with regression equation for Nile tilapia fed plant extracts.

The regression exponent of “ *b*” values in GLE-5 ( *b*=3.023, *t*(73)=39.216, *p*=0.01), GLE-10 ( *b*=3.101, *t*(73)=38.515, *p*=0.01), SGLE-5 ( *b*=3.067, *t*(73)=36.981, *p*=0.01), SGLE-10 ( *b*=3.003, *t*(73)=36.451, *p*=0.01), MxLE-5 ( *b*=3.135, *t*(73)=36.816, *p*=0.01), and MxLE-10 ( *b*=3.036, *t*(73)=33.390, *p*=0.01) were >3, indicating a positive allometric growth pattern in Nile tilapia compared to the control ( *b*=2.866, *t*(73)=33.881, *p*=0.01), which exhibited negative allometry. The length-weight correlation regression graphs for the control, GLE-5, GLE-10, SGLE-5, SGLE-10, MxLE-5, MxLE-10 diets are depicted in Figure 2. Raw data are available as underlying data (Tables 8-14). ^34^

### Condition factor

The condition factors were not significantly different at the end of the experimental period in the control ( *t*(148)=-0.599, *p*=0.275), GLE-5 ( *t*(148)=-0.199, *p*=0.421), GLE-10 ( *t*(148)=0.299, *p*=0.383), SGLE-5 ( *t*(148)=0.818, *p*=0.207), SGLE-10 ( *t*(148)=0.173, *p*=0.431), MxLE-5 ( *t*(148)=0.371, *p*=0.356), and MxLE-10 ( *t*(148)=-0.100, *p*=0.460) groups ( Table 2). Additionally, the minimum and the maximum values of condition factor for control, GLE-5, GLE-10, SGLE-5, SGLE-10, MxLE-5, and MxLE-10 groups were 0.865-1.177, 0.886-1.178, 0.824-1.097, 0.840-1.282, 0.817-1.162, 0.857-1.136, and 0.822-1.162, respectively. Raw data are available as underlying data (Tables 8-14). ^34^

### Correlation matrix for water quality and fish biometric parameters

The results of Pearson’s correlation analysis are presented in Table 3. A moderate positive correlation was observed between fish length and both ammonia (r = 0.403) and nitrite (r = 0.489), with the nitrite correlation being statistically significant at the 0.05 level ( Table 3). This finding suggests that as fish increase in length, there may be a corresponding rise in ammonia and nitrite levels, potentially due to metabolic byproducts. Similarly, fish weight exhibited a positive correlation with ammonia (r = 0.470) and nitrite (r = 0.489), with the latter correlation also significant at the 0.05 level. These results indicate that fish biomass may contribute to the elevation of these water quality parameters.

**Table 3.** Correlation matrix between water quality and fish biometric parameters.

Parameter	Length	Weight	Temperature	DO	pH	NH _**3**_-N	NO _**2**_-N	NO _**3**_-N
Length	1	0.969 ^**^	0.364	-0.199	-0.312	0.403	0.489 ^*^	-0.323
Weight	-	1	0.339	-0.215	-0.354	0.470 ^*^	0.489 ^*^	-0.258
Temperature	-	-	1	-0.684 ^**^	-0.563 ^**^	-0.060	0.426	-0.322
DO	-	-	-	1	0.887 ^**^	-0.185	-0.269	0.331
pH	-	-	-	-	1	-0.328	-0.385	0.326
NH _3_-N	-	-	-	-	-	1	-0.189	0.071
NO _2_-N	-	-	-	-	-	-	1	-0.207
NO _3_-N	-	-	-	-	-	-	-	1

** Correlation is significant at the 0.01 level (2-tailed).

* Correlation is significant at the 0.05 level (2-tailed).

Furthermore, a significant negative correlation (r = -0.684) was identified between temperature and dissolved oxygen at the 0.01 level, indicating that higher temperatures are associated with reduced dissolved oxygen levels. This phenomenon is well-documented, as warmer water typically holds less oxygen. Additionally, temperature demonstrated a moderate negative correlation with pH (r = -0.563), which was also significant at the 0.01 level. Elevated temperatures may influence the ionization of water, leading to decreased pH levels.

A strong positive correlation (r = 0.887) was observed between dissolved oxygen and pH, significant at the 0.01 level. Higher dissolved oxygen levels are often associated with elevated pH in aquatic systems, likely due to increased photosynthetic activity.

## Discussion

Aquafeed supplementation with herbal or plant extracts has proven to be an effective means of enhancing the growth performance, feed utilization, and immune response in farmed fish species. ^40^^,^^41^ The supplementation of natural bioactive rich-plant extracts may boost natural immunity and improve disease resistance, ^40^ resulting in higher production and profitability in the aqua industry. The length-weight relationship is used to predict the weight of fish in proportion to their length over a certain period. ^38^ Therefore, the present study investigated the effects of plant extract-supplemented diets on the length-weight relationship and condition factor of Nile tilapia.

The water quality parameters in all treatments were within the acceptable range, consistent with the findings of previous studies on spotted snakehead ( *Channa punctata*) under various feeding regimes involving agro-industrial by-products, ^42^ and on silver barb ( *Barbonymus gonionotus*) fingerlings fed with dietary blanched moringa leaf meal. ^43^ The specific growth rate showed a significant increase in all plant extract treatments. Similarly, the specific growth rate of spotted snakehead ( *C. punctata*) and silver barb ( *B. gonionotus*) was considerably improved under various feeding regimes involving agro-industrial by-products ^42^ and dietary blanched moringa leaf meal. ^43^

Aqua farmers and consumers prefer fish with isometric growth or body structure. ^27^^,^^44^ Furthermore, the length-weight relationship provides insights into the growth and health of a fish species. ^45^^,^^46^ In the present study, plant extract-supplemented diets observed significant correlation between length and weight of Nile tilapia. Our results are corroborated with previous study involving different maltose levels fed to Nile tilapia ( *O. niloticus*). ^27^ Importantly, Nile tilapia fed diets supplemented with GLE-5, GLE-10, SGLE-5, SGLE-10, MxLE-5, and MxLE-10 groups exhibited positive allometric growth pattern compared to the control. Similarly, silver barb ( *B. gonionotus*) and zebrafish ( *Danio rerio*) fed with dietary blanched moringa leaf meal ^43^ and plant-protein based diets ^47^ found positive allometric growth. On the contrary, gold fish ( *Carassius auratus*) fed with different concentrations of carrot ^48^ and gurami sago ( *Osphronemus goramy*) stocked in concrete ponds, floating net cages, and earthen freshwater ponds, ^49^ found negative allometric growth.

In the present study, plant extracts diets revealed a significant improvement in specific growth rate and positive allometric growth patterns of Nile tilapia. This can be ascribed to the presence of bioactive compounds in GLE and SGLE, including gallic acid, *p*-coumaric acid, quercetin, and kaempferol. These compounds have been demonstrated to enhance the growth of various fish species, including common carp *Cyprinus carpio*, ^50^ snakehead fish *Channa argus*, ^51^ grass carp *Ctenopharyngodon idellus,* ^52^ as well as certain animals. ^53^^,^^54^ Further research should be conducted in pond or cage culture systems to substantiate the positive allometric growth patterns exhibited by Nile tilapia when fed diets supplemented with GLE and SGLE.

The mean K _n_ values for both the plant extract diets and control were higher than one, indicating that the Nile tilapias were in good health condition during the experimental period. Similarly, condition factor values were more than one when Sargassum meal fed to Nile tilapia *O. niloticus*, ^31^ *Mansoa alliacea* hydroalcoholic extracts fed to pirarucu *Arapaima gigas*, ^55^ different maltose levels fed to Nile tilapia *O. niloticus.* ^27^

The strong correlations between fish length, weight, and specific water quality parameters, particularly ammonia and nitrite, suggest a potential interplay between fish growth and water quality dynamics. As fish grow, their metabolic waste production, including ammonia, increases, which can accumulate in the environment if not adequately managed. This accumulation of ammonia and nitrite can adversely affect water quality and fish health, ultimately impacting growth rates and overall well-being. Several studies support these observations. Elevated ammonia concentrations are known to impair fish growth by disrupting metabolic processes and diminishing feeding efficiency. For instance, in the rockfish *Sebastes schlegelii*, exposure to high ammonia concentrations resulted in reduced growth performance. ^56^ Similarly, increased nitrite levels have been associated with oxidative stress and compromised respiratory function in fish, which can further inhibit growth. ^57^ In aquaculture systems, higher fish densities or insufficient water treatment often exacerbate the accumulation of ammonia and nitrite. Such conditions have been shown to decrease feed consumption and utilization efficiency, as demonstrated in studies examining the effects of stocking density on water quality and fish performance. ^57^^,^^58^ These correlations underscore the necessity for meticulous management of water quality parameters in aquaculture to ensure optimal growth and health outcomes for fish populations.

## Conclusion

The specific growth rate was significantly higher in GLE, SGLE, and MxLE groups compared to the control group. The analysis of the regression equation indicated a positive correlation between the length and weight of Nile tilapia in the control group and the GLE, SGLE, and MxLE groups. Furthermore, the GLE, SGLE, and MxLE groups exhibited a positive allometric growth pattern in Nile tilapia compared to the control. The final condition factor values did not differ significantly between the control group and any of the GLE, SGLE, and MxLE groups. Consequently, these plant extracts demonstrate suitability for commercial Nile tilapia production.

## Data Availability

zenodo: Length-weight relationship and condition factor of Nile tilapia ( *Oreochromis niloticus*) fed diets supplemented with guava and star gooseberry leaf extract. https://doi.org/10.5281/zenodo.11174309. ^34^ This project contains the following underlying data:
-
Table 1: Weekly average temperature (°C) of the control, GLE-5, GLE-10, SGLE-5, SGLE-10, MxLE-5, and MxLE-10 groups. -
Table 2: Weekly average pH of the control, GLE-5, GLE-10, SGLE-5, SGLE-10, MxLE-5, and MxLE-10 groups. -
Table 3: Weekly average DO (mg/L) of the control, GLE-5, GLE-10, SGLE-5, SGLE-10, MxLE-5, and MxLE-10 groups. - Table 4: Weekly average NH _3_N (mg/L) of the control, GLE-5, GLE-10, SGLE-5, SGLE-10, MxLE-5, and MxLE-10 groups. - Table 5: Weekly average NO _2_N (mg/L) of the control, GLE-5, GLE-10, SGLE-5, SGLE-10, MxLE-5, and MxLE-10 groups. -
Table 6: Weekly average NO _3_N (mg/L) of the control, GLE-5, GLE-10, SGLE-5, SGLE-10, MxLE-5, and MxLE-10 groups. -
Table 7: Specific growth rate of Nile tilapia ( *N*=75) fed diets supplemented with control, GLE-5, GLE-10, SGLE-5, SGLE-10, MxLE-5, and MxLE-10 for 60 days. -
Table 8: Initial and final length (cm), weight (g), and condition factor of Nile tilapia ( *N*=75) fed diet supplemented with control for 60 days. -
Table 9: Initial and final length (cm), weight (g), condition factor of Nile tilapia ( *N*=75) fed diet supplemented with GLE-5 for 60 days. -
Table 10: Initial and final length (cm), weight (g), and condition factor of Nile tilapia ( *N*=75) fed diet supplemented with GLE-10 for 60 days. -
Table 11: Initial and final length (cm), weight (g), and condition factor of Nile tilapia ( *N*=75) fed diet supplemented with SGLE-5 for 60 days. -
Table 12: Initial and final length (cm), weight (g), and condition factor of Nile tilapia ( *N*=75) fed diet supplemented with SGLE-10 for 60 days. -
Table 13: Initial and final length (cm), weight (g), and condition factor of Nile tilapia ( *N*=75) fed diet supplemented with MxLE-5 for 60 days. -
Table 14: Initial and final length (cm), weight (g), and condition factor of Nile tilapia ( *N*=75) fed diet supplemented with MxLE-10 for 60 days. -
Table 15: Summary of the chemicals and reagents used in the experiment. - Authors checklist Manuscript No. 145369 (completed ARRIVE checklist). Data are available under the terms of the Creative Commons Attribution 4.0 International license (CC-BY 4.0).
