# Supplementary material for: Length-weight relationship and condition factor of Nile tilapia ( Oreochromis niloticus) fed diets supplemented with guava and star gooseberry leaf extract
Source: F1000Res. 2024 Oct 23;13:540. Originally published 2024 May 28. [Version 2] doi: 10.12688/f1000research.145369.2 (PMC11380074; doi:10.12688/f1000research.145369.2)
Supplement: Peer review history

# Reviewer report: Reviewer response for version 2

**DOI:** 10.5256/f1000research.173657.r362059

**Published:** 2025-01-30

**Authors:** Dr. Md. Abdullah-Al-Mamun

**Affiliations:** 1 Professor, Fish Health Management, Sylhet Agricultural University, Sylhet, Sylhet Division, Bangladesh

The manuscript is well-written, with a clear research objective and structured methodology. The study provides valuable insights into the effects of guava and star gooseberry leaf extract on the growth performance and condition factor of Nile tilapia. The findings, particularly the positive allometric growth patterns and improved specific growth rate, contribute to the field of aquaculture nutrition. However, the authors could extend their research by evaluating **disease resistance** to determine whether these plant extracts offer any significant benefits in enhancing the immune response of Nile tilapia. My recommendation is for **approval**, with this suggestion for future studies.

Is the work clearly and accurately presented and does it cite the current literature?

Yes

If applicable, is the statistical analysis and its interpretation appropriate?

I cannot comment. A qualified statistician is required.

Are all the source data underlying the results available to ensure full reproducibility?

Yes

Is the study design appropriate and is the work technically sound?

Yes

Are the conclusions drawn adequately supported by the results?

Yes

Are sufficient details of methods and analysis provided to allow replication by others?

Yes

Reviewer Expertise:

I specialize in aquaculture, finfish vaccination, aquaculture nutrition, fish immunology, and pathology. My research focuses on improving fish health and disease resistance through nutritional and immunological interventions.

I confirm that I have read this submission and believe that I have an appropriate level of expertise to confirm that it is of an acceptable scientific standard.

# Reviewer report: Reviewer response for version 1

**DOI:** 10.5256/f1000research.159308.r320437

**Published:** 2024-09-06

**Authors:** Agumassie Tesfahun

**Affiliations:** 1 Debre Tabor University, Debre Tabor, Ethiopia

I advice the authors to include correlation matrix or principal component analysis (PCA) regarding the water quality parameter and fish biometric relationships (length and weight). To this end, please refer other published works in the same title **. T**he paper is sound, however, it should be more sound if the authors include more statistical analysis such as correlation matrix or principal component analysis (PCA) to know the relationship between environmental variables and fish biometric data. Except for this issue, the paper is well presented and innovative for the scientific community.

 Capture fishery is now declining due to human induced impacts on the water bodies. Aquaculture is the alternative option to feed this fast-growing population. Therefore, this work is very important for the fish farmers and the community as well for food security.

Is the work clearly and accurately presented and does it cite the current literature?

Yes

If applicable, is the statistical analysis and its interpretation appropriate?

Partly

Are all the source data underlying the results available to ensure full reproducibility?

Yes

Is the study design appropriate and is the work technically sound?

Yes

Are the conclusions drawn adequately supported by the results?

Yes

Are sufficient details of methods and analysis provided to allow replication by others?

Yes

Reviewer Expertise:

Assistant Professor in Fisheries and Aquatic Sciences

I confirm that I have read this submission and believe that I have an appropriate level of expertise to confirm that it is of an acceptable scientific standard.

# Response

**Published:** 2024-10-17

**Authors:** nopadon pirarat

**Affiliations:** Veterinary Pathology, Faculty of Veterinary Science, Chulalongkorn University, Thailand

**Comment 1:** I advice the authors to include correlation matrix or principal component analysis (PCA) regarding the water quality parameter and fish biometric relationships (length and weight). ****To this end, please refer other published works in the same title **. **The paper is sound, however, it should be more sound if the authors include more statistical analysis such as correlation matrix or principal component analysis (PCA) to know the relationship between environmental variables and fish biometric data. Except for this issue, the paper is well presented and innovative for the scientific community.

 Capture fishery is now declining due to human induced impacts on the water bodies. Aquaculture is the alternative option to feed this fast-growing population. Therefore, this work is very important for the fish farmers and the community as well for food security.

**Response: **Thank you for your valuable feedback and appreciation of our work. We have incorporated a correlation matrix in the revised manuscript to analyze the relationships between water quality parameters and fish biometric data, specifically length and weight. This addition strengthens our analysis and aligns with your suggestion for more robust statistical evaluation.

 Your point regarding the declining capture fishery due to human-induced impacts is well taken. As aquaculture emerges as a vital alternative for sustaining the growing global population, our research aims to provide essential insights for fish farmers and contribute to food security efforts within communities.

 We appreciate your recognition of the significance of this work and look forward to any further suggestions you may have.

**In the revised manuscript**

**Correlation matrix for water quality and fish biometric parameters**

 The results of Pearson’s correlation analysis are presented in Table 3. A moderate positive correlation was observed between fish length and both ammonia (r = 0.403) and nitrite (r = 0.489), with the nitrite correlation being statistically significant at the 0.05 level (Table 3). This finding suggests that as fish increase in length, there may be a corresponding rise in ammonia and nitrite levels, potentially due to metabolic byproducts. Similarly, fish weight exhibited a positive correlation with ammonia (r = 0.470) and nitrite (r = 0.489), with the latter correlation also significant at the 0.05 level. These results indicate that fish biomass may contribute to the elevation of these water quality parameters.

 Furthermore, a significant negative correlation (r = -0.684) was identified between temperature and dissolved oxygen at the 0.01 level, indicating that higher temperatures are associated with reduced dissolved oxygen levels. This phenomenon is well-documented, as warmer water typically holds less oxygen. Additionally, temperature demonstrated a moderate negative correlation with pH (r = -0.563), which was also significant at the 0.01 level. Elevated temperatures may influence the ionization of water, leading to decreased pH levels.

 A strong positive correlation (r = 0.887) was observed between dissolved oxygen and pH, significant at the 0.01 level. Higher dissolved oxygen levels are often associated with elevated pH in aquatic systems, likely due to increased photosynthetic activity.

**Discussion**

 The strong correlations between fish length, weight, and specific water quality parameters, particularly ammonia and nitrite, suggest a potential interplay between fish growth and water quality dynamics. As fish grow, their metabolic waste production, including ammonia, increases, which can accumulate in the environment if not adequately managed. This accumulation of ammonia and nitrite can adversely affect water quality and fish health, ultimately impacting growth rates and overall well-being. Several studies support these observations. Elevated ammonia concentrations are known to impair fish growth by disrupting metabolic processes and diminishing feeding efficiency. For instance, in the rockfish *Sebastes schlegelii*, exposure to high ammonia concentrations resulted in reduced growth performance. ^56 ^Similarly, increased nitrite levels have been associated with oxidative stress and compromised respiratory function in fish, which can further inhibit growth. ^57 ^In aquaculture systems, higher fish densities or insufficient water treatment often exacerbate the accumulation of ammonia and nitrite. Such conditions have been shown to decrease feed consumption and utilization efficiency, as demonstrated in studies examining the effects of stocking density on water quality and fish performance. ^57, 58^ These correlations underscore the necessity for meticulous management of water quality parameters in aquaculture to ensure optimal growth and health outcomes for fish populations.

**References**

**56) **Shin KW, Kim SH, Kim JH, Hwang SD, Kang JC: Toxic effects of ammonia exposure on growth performance, hematological parameters, and plasma components in rockfish, *Sebastes schlegelii*, during thermal stress. Fish Aquat Sci. 2016;19:1–8. 10.1186/s41240-016-0044-6.

**57) **Al-Harbi AH, Siddiqui AQ: Effects of tilapia stocking densities on fish growth and water quality in tanks. Asian Fish Sci. 2000;13(4):391–396. 10.33997/j.afs.2000.13.4.011.

**58) **Sanders E, Farmer SC: Aquatic models: Water quality and stability and other environmental factors. ILAR J. 2019;60(2):141–149. 10.1093/ilar/ilaa008.

# Reviewer report: Reviewer response for version 1

**DOI:** 10.5256/f1000research.159308.r303303

**Published:** 2024-08-07

**Authors:** Dr.Sheikh Muhammad Azam

**Affiliations:** 1 Department of Zoology, Division of Science and Technology, Lahore, University of Education Lahore, Punjab, Pakistan

Work will attract the scientist in the field. The results will be helpful for farmers and aquaculturists.

Is the work clearly and accurately presented and does it cite the current literature?

Yes

If applicable, is the statistical analysis and its interpretation appropriate?

Yes

Are all the source data underlying the results available to ensure full reproducibility?

No

Is the study design appropriate and is the work technically sound?

Yes

Are the conclusions drawn adequately supported by the results?

Partly

Are sufficient details of methods and analysis provided to allow replication by others?

Yes

Reviewer Expertise:

fisheries, wildlife

I confirm that I have read this submission and believe that I have an appropriate level of expertise to confirm that it is of an acceptable scientific standard.

# Response

**Published:** 2024-10-17

**Authors:** nopadon pirarat

**Affiliations:** Veterinary Pathology, Faculty of Veterinary Science, Chulalongkorn University, Thailand

**Comment 1:** Work will attract the scientist in the field. The results will be helpful for farmers and aquaculturists.

**Response:** Thank you for taking the time to review our manuscript. We appreciate your positive feedback and are glad to hear that you believe our work will engage scientists in the field. We aim to provide valuable insights that will benefit both farmers and aquaculturists, contributing to more sustainable practices in aquaculture. Your encouragement reinforces the importance of our research, and we look forward to sharing our findings with the broader community.
